# Neurosarcoidosis Masquerading as Spinal Stenosis

**DOI:** 10.3390/diagnostics14202296

**Published:** 2024-10-16

**Authors:** Ameen Batheesh, Nina Borissovsky, Devy Zisman, Tal Gazitt

**Affiliations:** 1 Rheumatology Unit, Carmel Medical Center, Haifa 3436212, Israel; 2 Department of Radiology, Carmel Medical Center, Haifa 3436212, Israel; 3 The Ruth and Bruce Rappaport Faculty of Medicine, Technion-Israel Institute of Technology, Haifa 3109601, Israel

**Keywords:** neurosarcoidosis, spinal stenosis, trident sign, myelopathy, MRI

## Abstract

A 65-year-old woman was admitted to the neurology department with a suspected demyelinating disease due to complaints of progressive pain and weakness in both upper and lower limbs, as well as urinary incontinence. MRI of the spine revealed complex disc osteophyte with compression of the spinal cord in the cervical and lumbar spine at several vertebral levels, and localized enhancement in the cervical spine at the site of maximal spinal canal stenosis. During her hospitalization, the patient underwent extensive evaluation to rule out any systematic inflammatory diseases, infections, and malignancy. Chest CT revealed bilateral mediastinal lymphadenopathy. Transbronchial mediastinal lymph node biopsy showed numerous non-necrotizing granulomas without evidence of malignancy. After a thorough and careful exclusion of a demyelinating, infectious, and paraneoplastic myelopathies, and based on clinical, radiographic, and pathological findings, the patient was diagnosed with both neurosarcoidosis and spondylotic myelopathy. She was then treated for neurosarcoidosis, including glucocorticosteroids, azathioprine, and a biosimilar of the anti-TNF alpha agent infliximab, resulting in both clinical and radiographic improvement. Intramedullary spinal neurosarcoidosis is very rare and may present with clinical features of spondylotic myelopathy, with typical imaging findings occurring only in areas of spinal canal stenosis.

A 65-year-old female, with a known history of diabetes mellitus and hyperlipidemia, presented with 18 months of progressive gait instability, neck pain radiating to upper extremities, limb weakness, and new-onset urinary incontinence. Patient examination revealed bilateral proximal and distal muscle weakness, hyperreflexia in upper extremities, and hyporeflexia in lower extremities without sensory or cranial nerve involvement.

Blood tests were normal, including rheumatologic immune-serologic tests, Vitamin B12 level, creatine phosphokinase (CPK), angiotensin converting enzyme (ACE) level (<12), as well as infectious disease workup, including HIV, hepatitis B virus, hepatitis C virus, syphilis, brucella, bartonella henselae, Q-fever, toxoplasma, and toxocara.

Cerebrospinal fluid (CSF) analysis revealed a neutrophil-predominant white blood cell count (WBC) of 368 cells/μL, with an elevated protein level of 143 mg/dL (range, 15–45 mg/dL), normal glucose level 75 mg/dL, elevated IgG 24.1 mg/dL (range, 0.0–6.0 mg/dL), and was otherwise normal, including on pan-viral and pan-bacterial polymerase chain reaction (PCR) tests, CSF cultures, fluorescence-activated cell sorting (FACS) analysis, and tests for oligoclonal banding, anti-aquaporin-4, anti-myelin oligodendrocyte glycoprotein (MOG), and anti-neuronal antibodies.

Cervical spine magnetic resonance imaging (MRI) revealed complex disc osteophyte with compression of the spinal cord at C3-C7 with signal abnormality from C3-T3 (Figure 1, bracket in A) on T2 and STIR sagittal sequences. Post-gadolinium enhancement was seen on T1-weighted images at the dorsal and ventral subpial aspect of the cord (anterior myelitis with disc degeneration) [1,2] (Figure 1, arrow in B) with focal enhancing lesions at the area of canal stenosis. The enhancement extended to the central canal (TRIDENT SIGN) [3,4,5] (Figure 1, arrow in C) on axial post-gadolinium images. Notably, MRI of the lumbar spine showed degenerative changes with no signal abnormality in the lower spinal cord or within the spinal canal and MRI of the head was normal.

Chest computed tomography (CT) showed bilateral lymphadenopathy in the mediastinal and hilar regions. Transbronchial mediastinal lymph node biopsy revealed non-necrotizing granulomas without evidence of malignancy.

In accordance with the clinical, radiographic, and pathological findings and based on the 2018 Neurosarcoidosis Consortium Consensus Group diagnostic criteria [6], and after thorough and careful exclusion of a demyelinating, infectious, and paraneoplastic myelopathies, the patient was diagnosed with neurosarcoidosis (intramedullary spinal cord involvement) as well as spondylotic myelopathy.

The patient was treated with a 5-day pulse of intravenous (IV) methylprednisolone at dose 1000 mg followed by an oral prednisone taper with a starting dose of 60 mg/day, azathioprine 150 mg/day, and a biosimilar of the intravenous tumour necrosis factor (TNF)-alpha antagonist infliximab at a dose of 5 mg/kg every 8 weeks following an initial loading dose [7], resulting in marked improvement in cord signal abnormality (Figure 1, Image D) and decreased cord enhancement at 3 months’ follow-up imaging (Figure 1, Arrow in E).

Sarcoidosis involving the spine is uncommon, and intramedullary spinal cord involvement is even rarer, occurring in less than 1% of patients [8]. It may present with clinical features of spondylotic myelopathy, with typical imaging findings occurring only in areas of spinal canal stenosis [8], as in our case. The pattern of anterior myelitis with disc degeneration is an unusual and distinct finding observed on MRI in approximately 10% of patients with spinal cord involvement in sarcoidosis, and indicates the presence of two concurrent diseases (spinal cord sarcoidosis and spondylotic myelopathy) [1].

Although not pathognomonic, the trident sign is seen in ~9% of patients with spinal cord sarcoidosis, described as characteristic of sarcoidosis-associated myelopathy [3]. Accordingly, neurosarcoidosis should be considered in patients with cord enhancement centred at or just below a site of spinal canal stenosis.

## Figures and Tables

**Figure 1:**
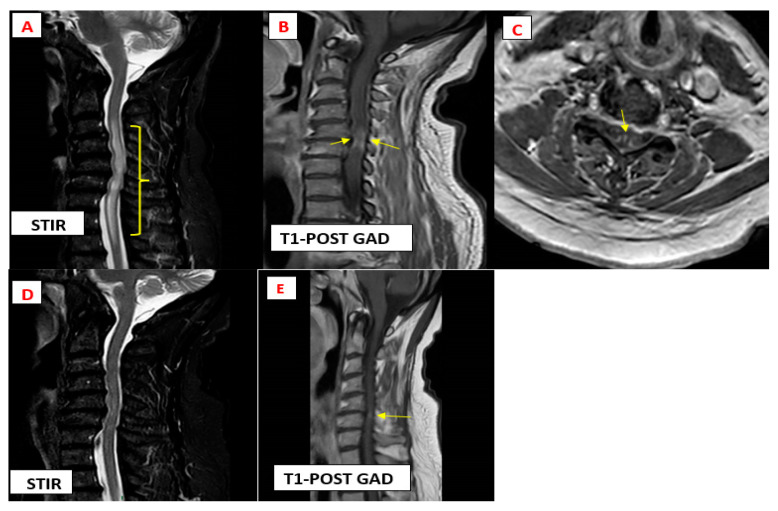
Magnetic resonance imaging findings typical of cervical myelopathy secondary to neurosarocidosis.

## Data Availability

All data supporting this publication are found within patient health records kept at Carmel Medical Center electronic medical records system.
